# Abstracts and Gleanings

**Published:** 1886-11-20

**Authors:** 


### ABSTRACTS AND GLEANINGS.

**The Excretion of Drugs by the Mammary Glands.**—A good many observations have been made upon the subject of the medicines excreted by the mammary glands. It has been claimed that mercury, iodine, bromine, arsenic, strychnine, sulphur, and chloroform may be thrown off by this gland when taken into the stomach of the nursing woman. It cannot be said, however, that our knowledge of the subject is as complete and definite as it should be, and hence, the recent experiments by Fehling are of interest. Fehling observed the effects on nursing, of various drugs given to nursing mothers. According to the Paris correspondent of the *British Medical Journal*, when doses varying from two to three grammes of salicylate of soda were administered to the nurse, every time that a child was suckled within an hour after the administration of the dose the salicylate appeared in its urine. After an interval of twenty-four hours there remained no trace of the drug. When the child was suckled too soon after the medicine had been taken, the salicylate could not be found in the urine. Elimination was completed at the same time in the mother and the child. With the iodide of potassium the results were the same. The milk, when analyzed, gave the characteristic reaction. In the infant, elimination lasted seventy-two hours, in the mother seventy-four. After twenty-four hours the milk still contained iodide of potassium. With ferrocyanide of potassium reaction was very pronounced in the maternal urine, but absent in the child's. Prolonged applications of iodoform upon vaginal and vulvar wounds of women in parturition, after prolonged use, generally resulted in iodine being found in the milk and urine of the mother, but not always in the urine of the infant. The child was never indisposed, even when iodoform was used to dry up the umbilical cord. There was only a small quantity of mercury transmitted through the milk of a nursing mother, and its presence was not constant. It appeared that the food of wet-nurses—even acid fruit juices and vinegar—had no influence on their nurslings. Thornhill had stated that he had observed prolonged sleep occur to children after administering to their wet-nurses such narcotics as tincture of opium in doses of from 20 to 25 drops. Fehling observed neither prolonged sleep nor constipation in the children. Hydrochlorate of morphine or chloral, in tolerable strong doses, did not affect the sucklings. Subcutaneous injections of moderately strong solutions of sulphate of atropine produced very pronounced symptoms in the mother, and dilatation of the pupil in the infant, which disappeared in twenty-four hours. This substance should, therefore, be employed in very feeble doses. In a very great majority of cases the milk of a woman attacked with fever had no influence on the nursling. In those rare cases where the temperature reaches 104°, the variations in the child's temperature were identical with those of the mother.

In some instances children had died of intestinal catarrh where the mother's milk could be the only cause of the affection. Bumm has observed, in a case of inflamed breast, the passage of the micrococci from the milk into the digestive apparatus of the child.—*Medical Record.*

**Another Application for Antipyrine.**—Dr. John Blake White, physician to Charity Hospital, New York, sends the following to the *New York Record:*

"The high road to truth is the knowledge of facts, and well it is for searchers after truth when facts can be ascertained and carefully recorded.

"Symptoms are the alphabet, cases the language, of disease, and that physician subserves his profession who carefully records his experience.

"During the past two years I have abundantly tested the therapeutic value of the drug known as antipyrine, in various forms of headache. The prompt relief obtained through its use compels me to accord to it a high rank among our medical resources. I have already called attention (*Medical News* July 10, 1886) to the potent antipyretc power possessed by this remedy in the management of various forms of fever, and have observed that after its administration the urgent symptom of headache, so uniformly present in these cases, was soon controlled.

"Antipyrine undoubtedly possesses bradycrote properties (*pulse retarding*) in a high degree, as the pulse is notably softened and moderated in frequency soon after a proper dose of it has been taken It produces some somatic change favorable to a reduction of the pulse in cases of fever, and so exerts a calming influence upon the vaso-motor system. The capillaries, through its agency, doubtless dilate, and local congestions are dissipated, as the relieved patient usually sinks into a refreshing repose soon after its exhibition. In the course of large experience with antipyrine I have found that, when administered in masterful doses, it not only promptly relieves the system of headache whenever present, whether resulting from disordered digestion, disturbance of the menstrual functions, loss of sleep, undue mental effort, or even associated with dreaded uræmia, but also possesses reliable prophylactic virtues against recurrent attacks of cranial neuralgia. So confident am I of the power of this remedy to disappoint neuralgic headache when imminent, that I have instructed many patients, who are liable to such visitations, to keep in readiness and take a dose of antipyrine as soon as they have premonition of its recurrenee, and all so far testify in favor of its efficacy.

"The value of this remedy in the above respect has not only been tested in my hospital and private practice, but I also record the fact that it has proved successful in the hands of professional friends, upon whom I had urged its employment for the relief of neuralgic affections of the head and face. I have been singularly impressed with the promptness of relief which often followed the administration of even a single dose of 15 grains of the antipyrine. The grateful relief from headache usually ensues within half an hour after the drug is taken. A sense of drowsiness ordinarily supervenes. followed by a brief but sufficient slumber, and the patient awakens quite relieved of this distressing symptom. I have never yet seen the sleep-disposing properties of antipyrine alluded to by any other observer, although this effect seldom fails to ensue when a full dose, such as I have named, has been taken."—*Medical Age.*

**Influence of Mental Impressions on the Fœtus.**—Fordyce Barker recently read a paper on this subject, at the American Gynecological Society's meeting He related the case of a young lady who was so profoundly affected by seeing Sothern play Lord Dundreary that her first boy, born four years later, exhibited peculiarities resembling those of his lordship.

Another case was that of a lady typically brunette; who married a typical blonde, but never was impregnated by him. After his death she married the "dark young man," a gentleman as marked a brunette as herself. Her first child was a decided blonde; the two subsequent born being brunettes.

The third case was that of a lady who, during the first month of pregnancy, had been much worried over her daughter having her ears pierced. When the babe was born both ears looked as if they had been pierced, and through one a thread could be passed.

The fourth case was that of a lady who, at an early period of pregnancy, was much impressed by seeing three ladies with hair-lips. When the child was born it had a double hair-lip.

A lady, married but a few weeks, was at the theatre with her husband. Something vexing him, he placed the point of his elbow on her hand, and held it so firmly that she fainted. The fingers were much swollen and painful for several days. She never lived with her husband afterwards. Two hundred and forty-eight days after this she gave birth to a son. On the left hand parts of all the digits were absent, as if they had been amputated.

[My case is even more remarkable than any of these. A lady during the early part of her pregnancy went one day into her pantry. The room was ill lighted, but she saw on a barrel a large, strange, black cat. In its efforts to escape the animal sprang directly in her face, where it fixed its claws. This was too much for the lady, and with one shriek of fear, she fainted. From that day to the end of her pregnancy she was possessed with an immovable conviction, that the results of her adventure would be manifested in the person of the child.

The day came, the poor mother went through the agonies of parturition, aggravated by her dread, and when the child was born, there wasn't anything abnormal about it!]—*Medical World.*

**Medical Men as Witnesses and Experts.**—Medical men are also citizens, and as often called upon to give testimony as any other class of the community in which they live, and if they are in possession of facts in connection with any case, they are required by law to reveal these facts just as any other witness is required to do so; but it is optional with the physician whether he plays the part of an expert or not. He can no more be required to give an *opinion* on medical or surgical points, than a lawyer can, without a fee, be required to give a legal opinion to the court. A physician may refuse to make an autopsy or chemical examination, or give an opinion in a hypothetical case without remuneration, just as a lawyer may refuse to give a legal opinion or advice without a fee. "The physician's opinion is the result of his education; his education, the result of his time and money expended in its acquirement," and is just as much his individual property as his medicine or his instruments; and a court has no more right to require him to give his opinion without pay, on the witness' stand, than the judge would have the right, on the street or in the physician's office, to require him to give an opinion and advice without a fee. This point has frequently been decided by the English courts. (See Carrington *vs.* Kerwan, N. P. 23; Webb *vs.* Page, March, J.) "It has been ruled that the testimony of an individual cited as an expert is voluntary, and he may decline to give it if it so seem fit to him."

Our attention has been called to this matter by the following decision of this point by Judge C. C. Fuller, of the State of Michigan: All honor to Judge Fuller!

EXPERT TESTIMONY.

Judge C. C. Fuller, of Mecosta County, in the case of "State of Michigan *vs.* Vanimmans," decided, when a physician refused to testify on the ground that the evidence would be expert testimony, "after many years' study and observation, I decide that a physician's knowledge is his stock in trade, his capital, and we have no more right to take it without extra compensation than we have to take provisions from a grocery, without pay, to feed the jury. The court rules that the witness is not compelled to testify."—*Southern Clinic.*

**Dysentery.**—Dr. Samuel H. Singleton (Mississippi Valley Medical Monthly) says: If called to a case of dysentery within twenty-four hours after the attack, and I find tormina and tenesmus very violent, which is generally the case, dejections mucus and bloody, I prescribe 3 grains of quinine every three hours, hot meal poultices to the bowels, and a tablespoonful of the following mixture every two hours:

℞ Sulphate magnesia.......................... ʒ iv,

Tincture opii........ .................. ʒ ijss,

Aromatic sulphuric acid........................ ʒ jss,

Water, add................................ ℥ iv. M.

Generally, after the third dose of this mixture has been given all of the above symptoms subside, the stools become serous and are passed without pain; in fact, a diarrhœa has been substituted for the dysentery. When, in spite of the above treatment, the bloody dejections continue, mixed with pus, tormina and tenesmus more frequent, a pill, given every two hours, of the following formula has wrought wonders in my hands:

℞. Pulverized opii, iodoform, aa..................gr. j,
Zinc sulphate..............................gr. ij. M.

I have treated twelve cases of dysentery with iodoform in the above combination—seven last season, and five this season—and my experience has been highly gratifying, every case having been pronounced out of danger or convalescent before the seventh day of treatment—a majority of them before the fifth day.—*American Medical Journal.*

**Treatment of Acute Rheumatism.**—Dr. T. C. Brown (Medical World) has the following to say on the above subject:

After a practice of a quarter of a century, I will now give the readers of the World my favorite treatment of acute rheumatism. On first seeing the case, if the bowels have not been well opened, I give a cathartic—say three compound cathartic pills, U. S. P.—and then order the following mixture:

℞. Pulvis potassii nitratis........................ʒ j,

Sodii salicylatis, potassi acetatis, aa............ʒ iv,

Extracti ergotæ fl., tincture aurantii, aa........flʒ iij,

Aquæ destillatæ, ad.........................fl℥ viij. M.

Sig. Tablespoonful every fourth hour.

When about the sixth dose has been taken the pain begins to leave. I continue on in the same way until all the pain is gone; then order the following:

℞. Pot. iod....................................ʒ j,

Vin. colch. rad., ext. sarsap. fl., aa..............fl℥ j,

Aq. dest., ad................................fl℥ viij. M.

S. Teaspoonful four times daily, and give one dose of the first mixture every night at bedtime while there is any pain.

The mixture No. 1 will surely relieve the pain, but if discontinued abruptly the pain will as surely return. One dose daily will usually keep pain abated; if not, a second for a time may be used.

Some may ask why the ergot is added to mixture No. 1. Any one who has had much experience with salicylic acid must have been perplexed with the ringing in the ears and head disturbance so often caused by its use. Now, my experience is that ergot will entirely remove or prevent the trouble. I am in love with salicylic acid, and prefer it in the form of salicylate of soda. Next to opium, I think it my favorite remedy.

The only local remedy I use in rheumatism is to keep the painful parts well wrapped in cotton batting, and if the joints are much swollen and skin tense, I bathe them occasionally with hot water. Since I have adopted the above treatment for rheumatism, I cannot think of a case where it has failed me, or one where there has been any heart lesion left behind.—*Ibid.*

**The Treatment of Gleet.**—In an address before the Medical Society of the county of Albany, Dr. O. D. Ball described a method of treatment employed by him successfully in a number of cases of chronic specific urethritis (Albany *Medical Annals*,

June 1886.) He employs an ointment composed of oxide of zinc, 3 ʒ; lard, 1 ℥ The application is made by means of an olive-pointed bougie. The constricted portion of the bougie is filled out evenly and smoothly as possible with the full calibre of the instrument. The bougie should be carried down to the prostatic portion of the urethra as rapidly as possible, and then, after being rotated in both directions, slightly withdrawn and pushed back again, in the hope that some of the ointment will be forced into the swollen mouths of the seminal and prostatic ducts. In the same manner the remaining portion of the urethra should be treated, giving plenty of time for the ointment to be melted and left in contact with the diseased membrane. The patient should have emptied his bladder previous to the application, and should be instructed to refrain from doing so again as long as possible. The applications should be made at least twice a day—in the morning and the last thing before retiring. The instrument should not be too large, but of just sufficient size to smooth out the folds of mucous membrane. For instance, when the penis measures three and a half inches in circumferance, a No. 20 French will about answer the purpose. The average time of treatment of all the cases was a little over four weeks. The longest any one case was under treatment was eight weeks; the shortest was ten days, except in one case where the patient never saw any discharge after the first application was made.—*Eastern Medical Journal.*

**Ptomaines.**—The ptomaine question is assuming importance. Ptomaines are cadaveric alkaloids—the results of putrefaction—and fish and meat poisonings are common, especially among users of canned goods. The food may be perfectly good when the can is opened, but after a can is opened a ptomaine may develop in a short time and render the food poisonous. These alkaloids were discovered by Armand Gautier, in 1870, and also by Selmi, just after, and are treated of in Dr. Clifford Mitchell's new book, The Physician's Chemistry. Gautier lately claimed that these bodies are constantly being formed in life, and that their non-elimination, or non-oxidation, is the cause of many diseases—thus opening up a new pathology, as well as dealing the so-called germ theory the severest blow it has yet received. Gautier's remarkable communication regarding ptomaines and leucomaines, made January 12, 1886, to the French Academy of Medicine (see Archives Generales des Medicine, No. 2, 1886), takes the ground that these by-products of normal vital action came through a putrefactive rather than combustive process, and says: "There would be a continual auto-infection from them if the skin, kidneys, bowels, and lungs did not act freely, and if the oxygen of the blood, which is their great enemy, were not continually supplied to the tissues." (This matter formed the subject of an editorial in the New York Medical Record, April 8, 1886, p. 392.) In July, 1884, I advanced the idea that many cases of peritonitis, septicæmia, puerperal fever, and analogous troubles, were caused by ptomaines. (Indianapolis News, July 14, 1884.) The fatal tyrotoxicon, from cheese, milk, picnic-ice-cream, is first cousin and is being watched.—*Med. Current.*

**Egg Diet.**—A patient had chronic diarrhœa of long standing and was much emaciated. The stomach, from taking a great many nauseous medicines (domestic practice stuff of "the-worse-it-is the-better-it-is" sort), had become so irritable that the patient was in danger of starving to death from inability to retain anything in the way of nutriment. All medication by the mouth was stopped and the patient was put upon a diet of raw albumen, prepared and administered thus: The whites of six raw eggs were beaten in a pint of cold water, a little sugar and flavoring added, and the mixture given *ad libitum*, to be taken by sips during the day. The number of eggs was gradually increased until it reached fifteen or eighteen per diem. In addition to this diet, the colon was filled once a day, by means of a gravity injecting apparatus, with a solution of carbolic acid (1 part of medicinal acid to 720 parts of water), which was allowed to escape immediately, no effort being made to retain it. The first two injections caused subsequent griping and tenesmus, which was relieved by rectal injections of laudanum and starch-water. The patient commenced to improve on the third day, and is now (five weeks later) entirely recovered, having gained sixteen pounds in weight in this time. A curious feature is, that the insipid albuminous drink, commenced from necessity is continued from choice, the patient having become quite fond of it—*Exchange.*

**Diphtheria.**—A writer in *Medical World* says: I desire to call the attention of the profession to a remedy that has been successful in my hands for a number of years, in the treatment of diphtheria; keeping in view, however, the proper nourishment and when necessary, stimulation of the patient, as well as the sanitary regulations of the sick-room. Leaving the chemical changes that follow in the mixing of the ingredients, out of the question; and by the way I would like to know the exact changes that do take place—I not only use it internally, but locally to the affected part, either as a gargle or by atomizer—I trust it will be given a trial, and will be anxious to hear whether it meets with the success that it has in my hands.

℞. Sodii hyposulph.............................℥ ss,

Tinct. iodi comp...............................f ʒ ss,

Tinct. ferri chloridi..............................f ℥ ss,

Spec tinct. aconit...............................f ʒ ss,

Aquæ destillat...............................f ℥ v,

Glycerin. puræ...............................f ℥ ij.

M. Sig.—One half to two teaspoonfuls every one, two or three hours according to height of fever, severity and age. At the same time using 2 teaspoonfuls as a gargle spray, every hour or two. When better, give the medicine internally, and use locally three or four times a day.

I have used the above not only in all stages of diphtheria, but in other affections of the throat.

I present a certain cure for that annoying skin disease—termed prairie itch, Wabash scratches, etc. I have never known it to fail when rightly used, to relieve the itching at once, and cure the trouble with several applications, besides being a fragrant lotion.

| | | |
|---|---|---|
| ℞. | Sodæ hyposulph............................ | ℥ ss, |
| | Chloral hydrat............................ | ʒ ij, |
| | Acid carbolic. cyst....................... | ʒ ss, |
| | Ol. picis................................. | fʒ j, |
| | Alcohol................................... | f℥ ss, |
| | Sp. myrciæ puræ........................... | f℥ ij, |
| | Glycerinæ................................. | f℥ jss, |
| | Aquæ...................................... | f℥ iv. |

Dissolve the soda and chloral in four ounces of water—dissolve the oil of tar and carbolic acid in alcohol, adding the bay rum and glycerin—mix both solutions together—apply night and morning to part involved, preceding the night application, with bathing in soap and water.

**An Anodyne for Use in Vesical Irritation.**—Dr. Wm. P. Copeland, of Eufaula, Ala., writes (Record): "In almost every community there are old men who suffer from enlarged prostates, accompanied with a chronic inflammation of the neck of the bladder, rendering them miserable sufferers, and a care and anxiety to their friends and families. Having had the professional care of several of this class of cases, and dreading the tendency they so frequently incur by the administration of opium for the relief of pain, I resorted to various washes for injecting the bladder, resulting in my adopting a solution of benzoate of soda, 10 grains to 1 ounce of water, with 20 to 30 drops of the green tincture of gelseminum. This is warmed, and injected by the patient through a soft rubber catheter whenever the pain is severe, and the catheter is then withdrawn, leaving the medicine to be voided in twenty or thirty minutes; or, where they are not able to pass anything from the bladder, the catheter is re-introduced and the medicine allowed to escape. My experience with this treatment has been so satisfactory that I cannot refrain from giving it publicity to the profession."—*Ib.*

**Treatment of Ulcerative Otorrhœa.**—With a mirror on his forehead, and a good light thrown down a silver speculum therewith, a surgeon first cleanses the whole meatus thoroughly by means of little rolls of salicylic wool, wrapped round the end of a tapering probe. If this is thoroughly done, the granulations will be seen in every part. Some of them may be so prominent as to form small polypi; others may be hardly at all raised. In either case, they must be scraped away freely with a sharp-edged curette and removed. The whole fundus of the ear is now cleansed and dried with small rolls of salicylic wool, as before; and, when quite dry, is touched freely with a roll of wool dipped in strong tincture of perchloride of iron. This fluid should be conveyed only to the ulcerating surface, and should be limited in amount. When it has remained in contact with the diseased area for a few seconds, it is dried off, and then a small quantity of iodoform, in fine powder, is blown over the part operated on. If this treatment be very carefully carried out in detail, ears which have been discharging for months or years may often be brought to heal in a few weeks. Everything depends on producing an aseptic, instead of a septic, condition in the ears.—*British Med. Journal.*

**Ten Commandments for Bathers.**—The following rules are from the *Rundschau* (Prag):

1. While suffering from violent mental excitement do not bathe.

2. While suffering from violent mental excitement do not bathe.

3. After sleepless nights or excessive exercise, do not bathe, unless you first rest a few hours.

4. After meals, and especially after taking alcoholic liquors, do not bathe.

5. Take your time on the way to the bathing or beach.

6. On arriving at the beach inquire about the depth and currents of the water.

7. Undress slowly, but then go at once into the water.

8. Jump in head first, or, at least, dip under quickly, if you do not like to do the first.

9. Do not remain two long in the water, especially if you are not very robust.

10. After bathing, rub the body to stimulate circulation, then dress quickly, and then take moderate exercise.—*National Drug.*

**Treatment for Diphtheria.**—Take equal parts (say 2 tablespoonfuls) of turpentine and liquid tar; put them into a tin pan or cup and set fire to the mixture, taking care to have a large pan under it as a safeguard against fire. A dense resinous smoke arises making the room dark. The patient immediately seems to experience relief; the choking and the rattle stop; the patient falls into a slumber and seems to inhale the smoke with pleasure; the fibrinous membrane soon becomes detached, and the patient coughs up microbes. These, when caught in a glass, may be seen to dissolve in the smoke. In the course of three days the patient *entirely* recovers.

Prescribed for many years by Sir James Bardsley, K. C. B., M. D., M. R. A. S., Inspector General of Hospitals for the Presidency of Bombay, India.

**Reflex Pain.**—Before the St. Louis Medico-Chirurgical Society (*St. Louis Courier of Medicine*), Dr. Todd said that frequently patients come with pain, and very considerable pain too, in the ear, generally confined to one ear, and upon examination there is no trouble found in the ear. Of course, in such a case the proper thing to do is to examine the mouth; and in all cases of pain about the ear, the mouth should be examined just as much as the ear itself.

A very intelligent gentleman, occupying a very important position as a city officer, came to him for treatment for pain in the ear. He examined him, and could find nothing the matter with the ear. He examined the mouth, and found one of the molar teeth on the same side with the painful ear which had been filled, and on striking it the tooth was sensitive. The doctor told him that in his opinion he did not have earache at all, but toothache, and that the pain in the ear was reflex; and to make sure that he wouldn't go away thinking that he did not know his business, he went down with him to a dentist and had him examine the tooth. He removed the filling, and immediately gas escaped, and the patient was re ieved of the pain in the ear. This is an illustration of the fact that the physician should have a good idea of dental troubles. The profession neglects them altogether too much, and trusts too much to the dentist.—*Medical and Surgical Reporter.*

**Turpentine as an Antidote to Phosphorus.**—The Paris correspondent of the *British Medical Journal* states that M. Ed. Rondot (Bordeaux), as the result of clinical observations and experiments, maintain the efficiency of turpentine in the treatment of poisoning by phosphorus, when taken either immediately or even some hours after the poison has been swallowed. The turpentine and phosphorus combine, and are eliminated without causing any other morbid phenomena than a local reaction on the alimentary and urinary organs. It is important to administer the turpentine at the outset, so as to neutralize the greatest quantity possible of the poison. Even if it be not completely neutralized, the oil of turpentine renders the symptoms milder and favors recovery. Turpentine diminishes hemorrhage and the nervous symptoms which follow poisoning by phosphorus.—*Ibid.*

**Squills in Whooping-Cough.**—M. Nettier, of Nancy, has treated a number of cases of whooping-cough with simple oxymel scillæ, which, he observes, ought to be ascertained to be good, as specimens to be found in pharmacies are of very different qualities. He advises the entire abandonment of cough syrups, and gives the squill even when there is diarrhœa and a good deal of vomiting. In the case of children who do not expectorate he even adds emetic drugs to the squill. He has seen cure result from this method of treatment in a few days, cases of failure being very rare. A colleague of M. Nettier, M. Remy, corroborates his statements, and says he has seen cases that have been going on for weeks yield to oymel scillæ in a few days.—*Lancet.*

**Piperine** is recommended as a valuable substitute for quinine in the treatment of intermittent fever, and it never affects the sensorium, though an equally good stimulant, carminative and febrifuge. It may be given without regard to the condition of the pulse or skin. Three grains may be given hourly for six hours at the onset; afterwards, when intermissiou is complete, the same dose every three hours; and when the paroxysms have been checked for some days, smaller doses, combined with blue pill and a little quinine.—*Ibid.*

